# Combined Structural and Computational Study of the mRubyFT Fluorescent Timer Locked in Its Blue Form

**DOI:** 10.3390/ijms24097906

**Published:** 2023-04-26

**Authors:** Konstantin M. Boyko, Maria G. Khrenova, Alena Y. Nikolaeva, Pavel V. Dorovatovskii, Anna V. Vlaskina, Oksana M. Subach, Vladimir O. Popov, Fedor V. Subach

**Affiliations:** 1 Bach Institute of Biochemistry, Research Center of Biotechnology of the Russian Academy of Sciences, Leninsky Prospekt. 33, bld. 2, 119071 Moscow, Russia; kmb@inbi.ras.ru (K.M.B.);; 2 Department of Chemistry, Lomonosov Moscow State University, Leninskie Gory 1/3, 119992 Moscow, Russia; 3 National Research Centre “Kurchatov Institute”, Kurchatov Complex NBICS-Technologies, Akad. Kurchatova sqr., 1, 123182 Moscow, Russiasubach_fv@nrcki.ru (F.V.S.)

**Keywords:** mRubyFT, mRuby, blue chromophore, fluorescent protein, cell timer, crystal structure, fluorescent timer, molecular modeling

## Abstract

The mRubyFT is a monomeric genetically encoded fluorescent timer based on the mRuby2 fluorescent protein, which is characterized by the complete maturation of the blue form with the subsequent conversion to the red one. It has higher brightness in mammalian cells and higher photostability compared with other fluorescent timers. A high-resolution structure is a known characteristic of the mRubyFT with the red form chromophore, but structural details of its blue form remain obscure. In order to obtain insight into this, we obtained an S148I variant of the mRubyFT (mRubyFT^S148I^) with the blocked over time blue form of the chromophore. X-ray data at a 1.8 Å resolution allowed us to propose a chromophore conformation and its interactions with the neighboring residues. The imidazolidinone moiety of the chromophore is completely matured, being a conjugated π-system. The methine bridge is not oxidized in the blue form bringing flexibility to the phenolic moiety that manifests itself in poor electron density. Integration of these data with the results of molecular dynamic simulation disclosed that the OH group of the phenolic moiety forms a hydrogen bond with the side chain of the T163 residue. A detailed comparison of mRubyFT^S148I^ with other available structures of the blue form of fluorescent proteins, Blue102 and mTagBFP, revealed a number of characteristic differences. Molecular dynamic simulations with the combined quantum mechanic/molecular mechanic potentials demonstrated that the blue form exists in two protonation states, anion and zwitterion, both sharing enolate tautomeric forms of the C=C–O^−^ fragment. These two forms have similar excitation energies, as evaluated by calculations. Finally, excited state molecular dynamic simulations showed that excitation of the chromophore in both protonation states leads to the same anionic fluorescent state. The data obtained shed light on the structural features and spectral properties of the blue form of the mRubyFT timer.

## 1. Introduction

Blue-to-red fluorescent timers (FTs) change their fluorescence color from blue to red during maturation [1,2,3]. Monomeric blue-to-red fluorescent timers Fast-FT [2], mRubyFT [3] and mTagFT [1] have been developed from the mCherry, mRuby2 and TagRFP red fluorescent proteins, respectively, and the corresponding mechanism of timer maturation with the formation of a red chromophore through its blue form has been proposed. To date, these are the only true timers, as others, pseudo-timers, have a mixture of different fluorescent forms, which mature independently and simultaneously [2,3]. True timers were used for the visualization of the LAMP2A protein trafficking and activation of the promoters [1], the labeling of the engram neuronal populations involved in two episodes of learning [1,4], the visualization of protein–protein interactions and the transitions between the phases of the cell cycle using the FucciFT2 system [1].

Structural data were obtained for the Fast-FT [5], mTagFT [1] and mRubyFT [3,6] timers. The spatial structure of the Fast-FT timer with a chemically degraded red chromophore was elucidated at a 1.8 Å resolution [5], which revealed the key role of R70, Y83 residues and the phenolic moiety of the chromophore in blue-to-red conversion. There was no electron density for the tyrosine ring of the red chromophore of the mTagFT timer, but information about the imidazolinone and acylimine part of the red chromophore was present and analysis of the chromophore’s immediate surroundings assisted in the understanding of its role in the adjustment of the spectral properties, maturation rates and stability of the blue form of the mTagFT timer [1]. The X-ray structure of the red form of the mRubyFT timer was obtained with the highest resolution for this type of timer and contained complete information about the red chromophore structure in its cis-configuration [3]. However, to date, information about the structure of the blue form of this timer has been obscured.

**Figure 1:**
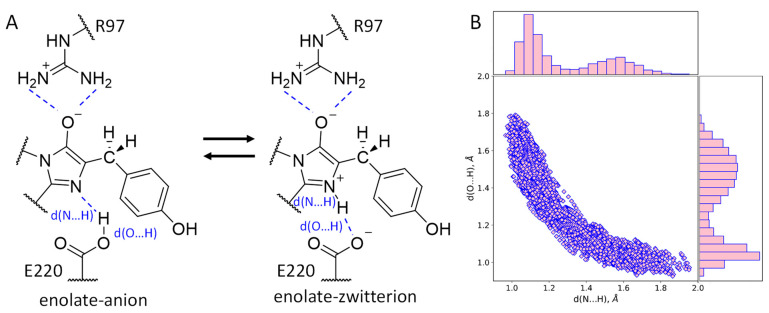
(**A**) Equilibrium between two protonation states, anionic and zwitterionic, of the enolate reaction intermediate obtained during the chromophore maturation after the dehydration step. (**B**) Distributions of the N…H and O…H distances of the hydrogen bonds between the chromophore and E220 (shown on panel (**A**)) obtained in the QM/MM MD trajectory.

According to experimental and computational studies, chromophore maturation in fluorescent proteins occurs in three steps: cyclization, dehydration and oxidation. After the dehydration step, an intermediate with the imidazolidinone ring and a reduced methine bridge is formed [7,8,9,10,11,12,13]. Still, it is not clear which particular protonation state of the chromophore intermediate exists as it is surrounded by two charged residues, E220 and R97 (Figure 1). Protonation states cannot be revealed directly from the X-ray data as this method does not resolve the positions of hydrogen atoms at a resolution worse than 1 Å, but they can be determined from molecular modeling.

Herein, we used a combination of conventional X-ray analysis and molecular modeling to study the structural details of the chromophore and its environment in the blue form of the mRubyFT timer. For this purpose, we constructed the S148I point mutant of the mRubyFT (here and after—mRubyFT^S148I^), which stabilizes the timer in its blue state over time. Structural analysis revealed that mRubyFT^S148I^ has a clear electron density for the majority of the protein. Still, this structure solved at 1.8 Å resolution revealed a lack of electron density for the phenolic moiety of the chromophore. We, however, were able to trace one conformation of this group with partial occupancy, which is somewhat similar to those found in the blue FP—mTagBFP [14]. A detailed analysis of the chromophore environment and its comparison with two related structurally characterized blue FPs—Blue102 and mTagBFP—revealed a number of characteristic differences. Finally, molecular dynamic simulation with classical and combined quantum mechanic/molecular mechanic (QM/MM) potentials complemented with the electron density analysis allowed us to detail the conformation of the chromophore phenyl moiety, determine the protonation state of the blue chromophore in the ground state and excited state minima regions.

## 2. Results and Discussion

### 2.1. mRubyFT^S148I^ Overall Structure and Chromophore Environment

**Figure 2:**
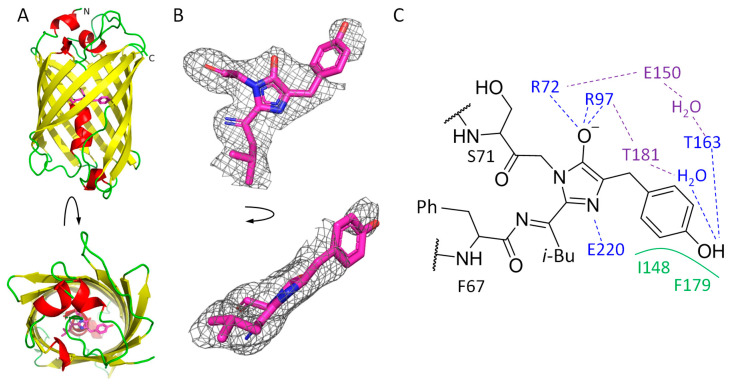
mRubyFT^S148I^ overall structure. (**A**) mRubyFT^S148I^ monomer colored in accordance with secondary structure. Chromophore is depicted in magenta. (**B**) Polder map at 3σ threshold around the chromophore calculated with Phenix [15]. Top (**left**) and side (**right**) views are depicted. (**C**) Chromophore and its immediate environment based on X-ray data: blue for hydrogen bonds with the chromophore, green for hydrophobic interactions with the chromophore and violet for hydrogen bonds in the chromophore-containing pocket.

The crystal structure of mRubyFT^S148I^ was elucidated using an X-ray crystallography method at 1.8 Å resolution (Figure 2A). There is one protein molecule per asymmetric unit, and contact analysis revealed that the protein is a monomer in the crystal. Introduced mutations did not alter the typical β-barrel fold of the protein. The chromophore positioned on the central helix of the barrel is formed by ^68^LYG^70^ amino acids (Figure 2A,B).

The chromophore is covalently bound to the neighboring F67 and S71 residues. Its imidazolidinone moiety is restricted on one side by the carboxyl oxygen of T65 and is additionally fixed by hydrogen bonds with R72, R97 and E220 (Figure 2C). The chromophore has a clear electron density except for its phenolic moiety, where density is poor (Figure 2B). This fact obstructed the unequivocal fit of the chromophore’s phenyl moiety, assuming the absence of its single conformation in all unit cells of the crystal. However, based on the density blob nearby (Figure 2B), at least one of the conformations can be modeled with partial occupancy, which is to some extent similar to the *trans* conformation found in the parent red protein, mRuby (PDB ID: 3U0L) [16]. In this conformation, the phenyl oxygen of the chromophore is directly hydrogen bound to the side chain of T163 as well as to E150 and T181 via a solvent water molecule. The overall structure of the imidazolidinone ring seems to be planar, suggesting that the conjugated π-system is formed. At the same time, the poor electron density of the phenyl moiety suggests that it could be flexible as the oxidation of the methine bridge did not occur. A poor electron density for the phenyl moiety of the chromophore is not unique and was found in some other FP structures, including mTagFT [1], mRubyFT^S148F^ [6] and GFPsol, a chemically reduced form of the GFP [7], where this fact was interpreted as a couple of conformations of the phenolic moiety.

The side chain of the I148 residue in mRubyFT^S148I^ has a clear electron density, with its side chain oriented towards the chromophore. Moreover, compared to the parental protein mRuby (corresponding residue H148), in mRubyFT^S148I^, isoleucine is shifted 1.3 Å closer (distance between Cα-atoms of corresponding residues) to the chromophore. This shift in the orientation of the I148 side chain hampers the rotation of the phenolic moiety of the chromophore to a *cis* conformation and restricts it from being coplanar with the imidazolidinone moiety. Instead, the angle between planes of phenolic and imidazolidinone rings is about 140° (Figure 2B).

### 2.2. Comparison with Blue102—A Blue Form of Fluorescent Timer Fast-FT

**Figure 3:**
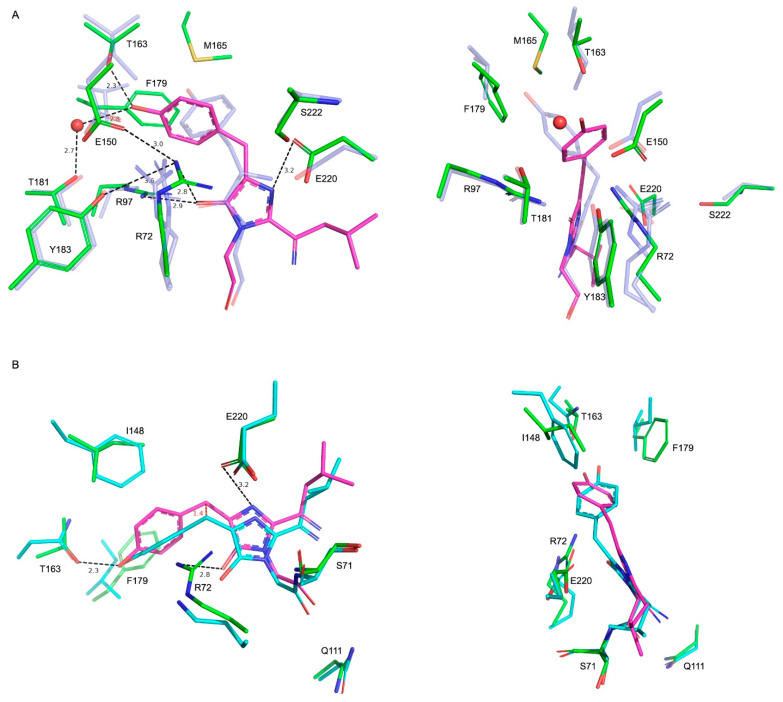
Structural comparison of mRubyFT^S148I^ with related FPs. (**A**) Superposition of the chromophore and environmental residues for mRubyFT^S148I^ (green) and Blue102 (semi-transparent blue) in two projections. Chromophore is shown in magenta for mRubyFT^S148I^. For clarity, hydrogen bond distances and labels are shown for mRubyFT^S148I^ only. (**B**) Superposition of the chromophore and environmental residues for mRubyFT^S148I^ (green) and mTagBFP (semi-transparent cyan) in two projections. Shift of the chromophore is indicated with red. Other colors and labels are similar to panel (**A**).

A number of blue FPs (BFP) have been described to date [14,17,18,19] as well as fluorescent timers that change their color from blue to red upon maturation [1,2,3,5]. A monomeric fluorescent timer, Fast-FT, made from mCherry FP, was previously structurally characterized together with its blue variant (Blue102, PDB ID—3LF4) with blocked blue-to-red conversion [5]. In addition, a similar orientation of a conserved R97, which fixes imidazolidinone carbonyl via a hydrogen bond, a structural comparison of mRubyFT^S148I^ and Blue102 revealed some important differences in the structure of the chromophore and its environment (Figure 3A). Firstly, in contrast to the degraded chromophore in Blue102, in mRubyFT^S148I^, there is a covalent bond between the imidazole and leucine moieties of the chromophore, indicating its stability in a crystal. In both structures, the phenolic moiety of the chromophore is non-coplanar to the imidazolic one and has a single CA2-CB2 bond based on a small valence angle; however, the positions of tyrosine moieties differ (Figure 3A). Compared to Blue102, in mRubyFT^S148I^, the phenolic moiety seems to be flipped about the CB2 atom, which leads to the reorientation of this group towards R72, whose side chain introduces steric hindrances to a phenolic moiety of the chromophore, restricting its rotation around the CB2-CG2 bond. In mRubyFT^S148I^, side chains of M165 (Q163 in Blue102) and F179 (V177) extrude the phenolic moiety from the orientation found in Blue102. Moreover, in mRubyFT^S148I^, the side chain oxygens of T163 (I161) and T181 (T179) make hydrogen bonds to the OH group of the chromophore, additionally fixing the phenolic moiety orientation.

The R72 side chain of mRubyFT^S148I^ has only one conformation, whose guanidine part is almost perpendicular to those of both conformations of the corresponding R70 of Blue102. Despite that, in both structures, these residues share a similar hydrogen bonding network to imidazolon oxygen as well as to the OH-group of Y183 (Y181) and the side chain of E150 (E148).

Finally, the side chain of E220 in mRubyFT^S148I^ forms a relatively weak hydrogen bond to imidazolidinone nitrogen of the chromophore (corresponding distance is 3.2 Å) due to the rotation of the former at about 90° compared to the corresponding E215 in Blue102, where this bond is stronger (distance is 2.4 Å) due to the rotated side chain. The E220 side chain also lacks a direct hydrogen bond to S222 (A217), while in the Fast-FT timer, similar residues (E215 and S217) interact via a hydrogen bond. However, in Blue102, this interaction is also absent due to the substitution of the corresponding serine for alanine.

### 2.3. Comparison with mTagBFP

In addition to Blue102, the second known structure of blue FP with identical residues forming chromophores is mTagBFP (PDB ID—3M24) [20]. We compared the structure of mTagBFP with mRubyFT^S148I^. In mTagBFP, the chromophore also occupies a *trans* conformation (Figure 3B) with the imidazolidinone moiety of the chromophores sharing a similar coordination via hydrogen bonds to E220 (E215 in mTagBFP) and R72 (K67) and the OH group forming a hydrogen bond with the side chain of T163 (N158). The chromophore conformation differs between these two structures, with a rotation around the C1-N3 bond at about 20˚ accompanied by a shift of the chromophore towards the R72 (K67) residue up to 1.4 Å (distance between corresponding CB2 atoms). Finally, the phenolic moiety of the mRubyFT^S148I^ chromophore is rotated at about 50˚ compared to those in mTagBFP. Three substitutions in the vicinity of the phenolic moiety seem to be the cause of such chromophore reorientation in the case of mRubyFT^S148I^. These are I148 (F143 in mTagBFP), F179 (I174) and R72 (K67), which sterically restrict the position and orientation of the phenolic moiety (Figure 3B). Another difference between structures is the conformation of the leucine moiety, which is a part of the chromophore. Compared to mTagBFP, in mRubyFT^S148I^, this moiety is rotated at about 90° and resembles that in the structure of Blue102. The other important residues for chromophore maturation residues—S71 (S66), Q111 (Q106) and E220 (E215)—have similar conformations in both proteins.

### 2.4. Structure and Protonation State of the Chromophore in the mRubyFT^S148I^

We started with classical MD simulations to properly locate the phenyl moiety of the chromophore. Its conformation, reconstructed based on the X-ray data, remained stable during the entire simulation: the stable hydrogen bond between the OH groups of the phenyl fragment of the chromophore and T163 remained. The representative frame from the MD simulation was utilized in the following QM(PBE0-D3/6-31G**)/MM(CHARMM) MD simulations to study in detail the properties of the imidazolidinone fragment that is responsible for the photophysical properties of the blue form of mRubyFT^S148I^.

The first issue to be considered is the protonation state of the imidazolidinone moiety (Figure 1A). Its carbonyl oxygen atom forms hydrogen bonds as an acceptor with the side chain of R97. A nitrogen atom of the imidazolidinone moiety located on the hydrogen bond distance from the carboxylate of the E220 and a proton should be present between these two atoms. Still, it is not evident which of these heteroatoms acts as a donor and which as an acceptor of the hydrogen bond. To clarify this, we started the QM/MM MD simulation of the structure with the hydrogen atom at a covalent bond distance from the nitrogen atom of the chromophore. During the 10 ps production run, the equilibrium between the N–H…O (zwitterionic chromophore) and N…H–O (anionic chromophore) states of the hydrogen bond between the chromophore and E220 was observed (Figure 1). The corresponding distributions of N…H and O…H distances revealed that both conformations are almost equally populated (Figure 1B). The distance between heavy atoms, N and O, is short during the entire simulation, not exceeding 2.90 Å; it is described by a normal distribution with a mean value of 2.58 Å and 0.08 Å standard deviation. Thus, the hydrogen bond between the chromophore nitrogen atom and E220 is strong, and its existence in both the N–H…O and N…H–O states indicates that the pK_a_ values of the nitrogen atom in a blue chromophore and the carboxylate oxygen of the E220 residue are similar. In fluorescent proteins with a completely matured chromophore, this hydrogen bond exists in the N…H–O state [21,22]. Therefore, we can deduce that in the matured chromophore the nitrogen atom is more acidic.

**Figure 4:**
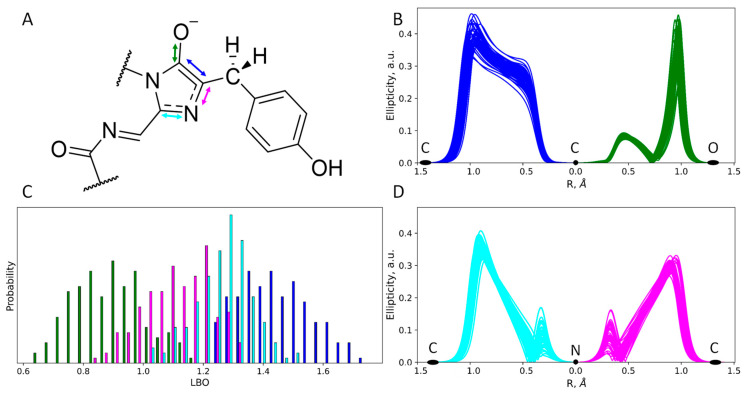
Structure and electron density features of the anionic imidazolidinone moiety of the mRubyFT^S148I^ chromophore. (**A**) Chemical structure of the chromophore revealed from the electron density analysis. Mean distances and standard deviations obtained in the QM/MM MD trajectory are in Å. (**B**,**D**) Ellipticity curves along covalent bonds from the imidazolidinone moiety depicted for 50 QM/MM MD frames. (**C**) Distributions of the Laplacian bond order values. For colors attributed to certain bonds on panels (**B**–**D**) see arrow colors on panel (**A**).

**Figure 5:**
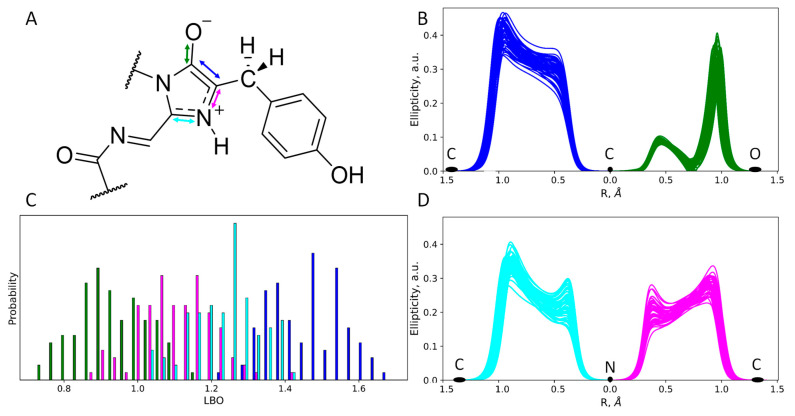
Structure and electron density features of the zwitterionic imidazolidinone moiety of the mRubyFT^S148I^ chromophore. (**A**) Chemical structure of the chromophore revealed from the electron density analysis. Mean distances and standard deviations obtained in the QM/MM MD trajectory are in Å. (**B**,**D**) Ellipticity curves along covalent bonds from the imidazolidinone moiety depicted for 50 QM/MM MD frames. (**C**) Distributions of the Laplacian bond order values. For colors attributed to certain bonds on panels (**B**–**D**) see arrow colors on panel (**A**).

To further study the chemical structure of the chromophore in both protonation states, we performed an electron density analysis, including Laplacian bond order (LBO) determination and ellipticity analysis along the covalent bonds (Figure 4 and Figure 5). Despite the different protonation states of the imidazolidinone moiety, the O^−^–C=C fragment exists in the enolate form that is seen from both LBO indices (Figure 4C and Figure 5C) and ellipticity calculated along the corresponding covalent bonds (Figure 4B and Figure 5B). Ellipticity curves along the neighboring C–N bond differ depending on the protonation state of the chromophore (Figure 4D and Figure 5D). For the chromophore in the anionic form (Figure 4D), the ellipticity curves demonstrated a higher concentration of the negative charge on the carbon atoms; that is, C–N bonds are polarized to a higher extent. For the neutral species, the polarization is less pronounced and the bond order is generally higher, as seen from the flat region between the nitrogen and carbon atoms, with the ellipticity being around 0.2 a.u.

### 2.5. Photophysical Properties of the Chromophore: Experiment and Calculations

**Figure 6:**
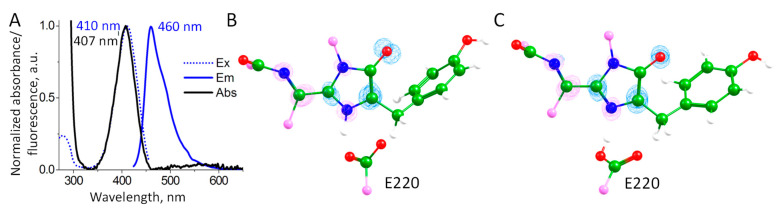
(**A**) Absorption, excitation and emission spectra of the mRuby^S148I^. (**B**,**C**) Electron density redistribution upon excitation from the S_0_ state to the S_1_ state. Blue and magenta isosurfaces correspond to the decrease and increase in electron density upon excitation, respectively. The isovalues are +/–0.0065 a.u. (**B**) A chromophore in the zwitterionic state; (**C**) a chromophore in the anionic state. Color code: carbon—green; nitrogen—blue; oxygen—red; hydrogen—white; carbon atoms on the border of the depicted molecular fragment—pink.

We studied the photophysical properties of mRubyFT^S148I^ including the absorption, excitation and emission spectra (Figure 6A). The absorption/excitation/emission band maxima at 407/410/460 nm are within a 3 nm difference from the blue form of the mRubyFT timer, indicating that the S148I substitution practically did not affect the photophysical properties of the blue chromophore but blocked the transition into the red form.

The QM(TD-ωB97X-D3/6-31G**)/MM(CHARMM) calculations were performed to evaluate S_0_–S_1_ excitation energies at 100 QM(PBE0-D3/6-31G**)/MM(CHARMM) MD frames for each protonation state of the chromophore. We obtained distributions of the excitation energies with similar mean values: 4.05 ± 0.09 eV for the chromophore in the anionic state and 3.94 ± 0.13 eV for the zwitterionic state. The calculated electron density redistribution is the same for a chromophore in both protonation states (Figure 6B,C). It is characterized by the decrease in the electron density on the carbon atom of the enolate fragment and an increase in the nitrogen atom and the extended conjugated π-system outside of the imidazolidinone ring. This is in line with the electron density redistribution upon excitation for the GFP-type chromophore and its analogs with the extended π-system [23,24]. Thus, both protonation states of the chromophore may contribute to the observed absorption band.

We extracted representative frames corresponding to both protonation states of the chromophore and performed the QM(TD-ωB97X-D3/6-31G**)/MM(CHARMM) MD simulation at the first excited singlet state, S_1_. After ~300 fs of simulation, we achieved an S_1_ minimum region that turned out to be anionic for both systems. Thus, in both populations with different protonation states of the chromophore, the anionic state is responsible for the fluorescence.

## 3. Material and Methods

### 3.1. mRubyFT^S148I^ Cloning, Expression and Purification

The preparative mRubyFT^S148I^ protein expression and purification for structural studies were performed as described earlier [1]. Briefly, the gene of the mRubyFT^S148I^ protein was amplified via PCR and inserted at BglII/EcoRI restriction sites of the pBAD/HisB-TEV plasmid. The plasmid was transformed into BW25113 bacterial cells using chemical transformation. The overnight cultures with bacterial cells expressing the mRubyFT^S148I^ with the N-terminal His-tag and tobacco etch virus (TEV) protease cleavage site were centrifuged for 20 min at 5000 rpm at 4 °C (Beckman Coulter centrifuge, Brea, CA, USA). The pellet was then resuspended in 100 mL of buffer A (40 mM Tris-HCl, pH 7.8, containing 400 mM NaCl and 10 mM imidazole), supplemented with 0.2% Triton X-100 and 1 mM phenylmethylsulfonyl fluoride, and sonicated using the following conditions: pulse 2 s, pause 6 s, amplitude 45%, total time 5 min. After centrifugation for 30 min at 28,000× *g*, at 4 °C (Beckman Coulter centrifuge, Brea, CA, USA), the supernatant was loaded onto a 5 mL Ni-NTA Superflow column (Qiagen, Hilden, Germany). Successive washes were then performed with buffer A and buffer A supplemented with 40 mM imidazole. Protein was eluted with buffer A supplemented with 300 mM imidazole. After the addition of 1 mM DTT and 1 mM EDTA, the His-tag was cleaved from the protein using digestion with TEV protease (1 mg per 10 mg of protein); the digest was dialyzed for 16 h in buffer B (40 mM Tris, pH 7.8, 400 mM NaCl, 5 mM imidazole, 2 mM BME, 1 mM EDTA) at +4 °C. The digested protein was then loaded onto a Ni-NTA Superflow column (Qiagen, EU) equilibrated with buffer B; TEV protease and cleaved His-tag were bound to the Ni-NTA Superflow column (Qiagen, EU), the fused protein was concentrated using a 10 kDa cutoff concentrator (Millipore, Burlington, MA, USA) and loaded onto a HiTrap Desalting column (GE Healthcare, Danderyd, Sweden) in 50 mM Na-phosphate buffer. The protein was further purified using a MonoS column (GE Healthcare, Danderyd, Sweden) equilibrated with 50 mM phosphate buffer at pH 7.0. Protein was eluted using a linear gradient of NaCl concentration. The fractions containing the target protein were combined, concentrated using 10 kDa cutoff concentrators (Millipore, Burlington, MA, USA) and transferred to 20 mM Tris buffer pH 8.0, 150 mM NaCl on a PD-10 column (GE Healthcare, Danderyd, Sweden). Protein concentration was then measured by the Bicinchoninic Acid Protein Assay Kit (Sigma-Aldrich, Saint Louis, MO, USA). P0914-5AMP solution (Sigma-Aldrich, Saint Louis, MO, USA) was used as the BSA protein standard. The purity of the preparations at all stages was monitored by electrophoresis in PAGE (gel concentration 15%). Protein chromatography was performed using ÄKTA prime plus and ÄKTA explorer 100 systems (GE Healthcare, Danderid, Sweden).

### 3.2. Spectral Characterization

Absorption and fluorescence spectra of the protein were recorded using an SM2203 spectrofluorometer (Solar, Minsk, Belarus) and a NanoDrop 2000c spectrophotometer (Thermo Scientific, Waltham, MA, USA), respectively.

### 3.3. Crystallization and Structure Determination of mRubyFT^S148I^

An initial crystallization screening of mRubyFT^S138I^ was performed with a robotic crystallization system (Rigaku, USA) and commercially available 96-well crystallization screens (Hampton Research, Aliso Viejo, CA, USA and Anatrace, Maumee, OH, USA) at 15 °C using the sitting drop vapor diffusion method. The protein concentration was 15 mg/mL in the following buffer: 20 mM Tris-HCl, 200 mM NaCl, pH 7.5. Optimization of the initial conditions was performed by the hanging-drop vapor-diffusion method in 24-well VDX plates. Crystals suitable for data collection were obtained under the following conditions: 0.1 M Bis-tris pH 5.5, 19% PEG 3350.

mRubyFT^S148I^ crystals were briefly soaked in 100% Paratone oil (Hampton research, Aliso Viejo, CA, USA) immediately prior to diffraction data collection and flash-frozen in liquid nitrogen. The X-ray data were collected from a single crystal at 100 K at the beamline “Belok-RSA” of the Kurchatov SNC (Moscow, Russia). The data were indexed, integrated and scaled using Dials program [25] (Table 1). The program Pointless [26] suggested orthorhombic space group P2_1_2_1_2_1_.

The structure was solved by the molecular replacement method using MOLREP program [27] and the structure of the mRuby FP (PDB ID 3U0L) as an initial model. The refinement of the structure was carried out using Refmac5 [28] and BUSTER [29], implemented in the CCP4 suite [30]. The visual inspection of electron density maps and the manual rebuilding of the model were carried out using the COOT interactive graphics program [31]. The resolution was successively increased to 1.8 Å and the hydrogen atoms in fixed positions and TLS were introduced during the final refinement cycles. In the final model, an asymmetric unit contained one independent copy of the protein of 224 residues, including the chromophore and 76 water molecules. The first two residues from the N-terminal as well as ten residues from the C-terminal part of the protein were not visible in electron density, possibly due to high flexibility. Structure validation revealed that 97.2% of residues are in the most favored regions of Ramachandran plot with additional 2.8% in allowed regions, indicating non-strained geometry.

### 3.4. Structural Analysis

Structural analysis was performed using PDBePISA [32] and PDBeFOLD [33] services. Figures were made with PyMol (The PyMOL Molecular Graphics System, Version 1.3 Schrödinger, LLC, New York, NY, USA).

### 3.5. Molecular Modeling

The full-atom 3D model of mRubyFT^S147I^ was obtained from the elucidated crystal structure. The CHARMM36 [34,35] force field parameters were utilized for protein and the CGenFF [36] force field parameters for the chromophore. The system was solvated in the rectangular water box with the TIP3P [37] water molecules and neutralized. Classical molecular dynamic simulations were performed in the NAMD3 software package [38]. The system was preliminarily equilibrated by 10,000 minimization steps and 50 ns MD run in the NPT ensemble at p = 1 atm and T = 300 K. The pressure and temperature were controlled by Nosé–Hoover barostat and Langevin thermostat, respectively. Equilibrated system was utilized for the subsequent molecular dynamics simulations with the combined quantum mechanics/molecular mechanics potentials. The MM subsystem was described with the classical force fields described above, and the QM subsystem was described at the density functional theory (DFT) level with the hybrid functional PBE0 [39] with the D3 [40] dispersion correction and 6-31G** basis set. The QM subsystem included the chromophore and side chains of the neighboring residues, R72, R97, E150, T163, H202, E220 and two water molecules. The 10 ps equilibration QM/MM MD run was followed by the 10 ps production run. The QM/MM MD simulations were performed using the interface [41] for the classical molecular dynamics software NAMD2 and the quantum chemistry package TeraChem [42]. Two protonation states of the chromophore were found along the trajectories. We selected sets of 100 frames for each state and performed electron density analysis using Multiwfn program [43]. Laplacian bond order (LBO) indices [44] were calculated at each frame for the imidazolidinone fragment. Ellipticity [45] profiles were calculated along covalent bonds of the same fragment of the chromophore. For these frames, we also calculate vertical S_0_–S_1_ excitation energies at the TDDFT level with the ωB97X-D3 functional [46] and 6-31G** basis set using ORCA 4.2.1 software [47]. Additional QM(TD-ωB97X-D3/6-31G**)/MM MD simulations at the S_1_ state were performed starting from both conformations to locate the S_1_ minimum region corresponding to the fluorescence.

## 4. Conclusions

We utilized a combination of structural and computational biology methods for a comprehensive study of the S148I variant of the mRubyFT blocked in its intermediate maturation state with the blue form of the chromophore. X-ray data at a 1.8 Å resolution revealed that the methine bridge of the chromophore is not oxidized in this state, thus bringing conformational flexibility to the phenolic moiety of the chromophore, which was deduced from the poor electron density. Molecular dynamic simulations demonstrated that in the most populated conformation, the phenolic moiety is fixed by the hydrogen bond with the side chain of the T163 residue. Additionally, QM/MM MD simulations showed that the chromophore exists in the enolate tautomeric state and that the equilibrium between two protonation states with respect to the nitrogen atom of the imidazolidinone ring exists. Both of these states have similar calculated S_0_–S_1_ vertical excitation energy values and can be attributed to the excitation band maximum at 410 nm. Excited-state molecular dynamics demonstrated that excitation of the chromophore in both protonation states leads to the same anionic fluorescent state observed in the experimental fluorescence spectrum, with the band maximum at 460 nm.

## Figures and Tables

**Table 1:** Data collection, processing and refinement.

Data Collection	
Diffraction source	”Belok-RSA“ beamline, NRC “Kurchatov Institute”
Wavelength (Å)	0.79
Temperature (K)	100
Detector	CCD
Crystal-to-detector distance (mm)	120.00
Rotation range per image (°)	1.0
Total rotation range (°)	130
Space group	P2_1_2_1_2_1_
a, b, c (Å)	31.79; 66.83; 97.76
α, β, γ (°)	90.0; 90.0; 90.0
Unique reflections	19640 (1116)
Resolution range (Å)	48.9–1.80 (1.84–1.80)
Completeness (%)	98.2 (96.7)
Average redundancy	4.9 (5.2)
〈I/σ(I)〉	6.1 (0.2)
Rmeas (%)	9.0 (133.0)
CC_1/2_	100.0 (51.0)
**Refinement**	
*R_fact_ (%)*	22.7
*R*_free._ *(%)*	26.8
Bonds (Å)	0.01
Angles (°)	2.04
*Ramachandran plot*	
Most favored (%)	97.2
Allowed (%)	2.8
*No. atoms*	
Protein	1741
Water	76
Chromophore	23
Other ligands	0
*B-factors (Å* ^2^ *)*	
Protein	29.5
Water	37.1
Chromophore	38.8
**PDB ID**	**7Q6B**

Values in parenthesis are for the highest-resolution shell.

## Data Availability

The refined model and structure factors have been deposited in the Protein Data Bank under the accession code 7Q6B. All materials are available from the corresponding author upon reasonable request.

## References

[1] Subach O.M., Vlaskina A.V., Agapova Y.K., Nikolaeva A.Y., Anokhin K.V., Piatkevich K.D., Patrushev M.V., Boyko K.M., Subach F.V. (2023). Blue-to-Red TagFT, mTagFT, mTsFT, and Green-to-FarRed mNeptusFT2 Proteins, Genetically Encoded True and Tandem Fluorescent Timers. Int. J. Mol. Sci..

[2] Subach F.V., Subach O.M., Gundorov I.S., Morozova K.S., Piatkevich K.D., Cuervo A.M., Verkhusha V. (2009). V Monomeric fluorescent timers that change color from blue to red report on cellular trafficking. Nat. Chem. Biol..

[3] Subach O.M., Tashkeev A., Vlaskina A.V., Petrenko D.E., Gaivoronskii F.A., Nikolaeva A.Y., Ivashkina O.I., Anokhin K.V., Popov V.O., Boyko K.M. (2022). The mRubyFT Protein, Genetically Encoded Blue-to-Red Fluorescent Timer. Int. J. Mol. Sci..

[4] Okuyama T., Kitamura T., Roy D.S., Itohara S., Tonegawa S. (2016). Ventral CA1 neurons store social memory. Science.

[5] Pletnev S., Subach F.V., Dauter Z., Wlodawer A., Verkhusha V.V. (2010). Understanding Blue-to-Red Conversion in Monomeric Fluorescent Timers and Hydrolytic Degradation of Their Chromophores. J. Am. Chem. Soc..

[6] Boyko K.M., Nikolaeva A.Y., Dorovatovskii P.V., Vlaskina A.V., Subach O.M., Subach F.V. (2022). Three-Dimensional Structure of the S148F Mutant of Blue-to-Red Fluorescent Timer mRubyFT. Crystallogr. Reports.

[7] Barondeau D.P., Tainer J.A., Getzoff E.D. (2006). Structural Evidence for an Enolate Intermediate in GFP Fluorophore Biosynthesis. J. Am. Chem. Soc..

[8] Heim R., Cubitt A.B., Tsien R.Y. (1995). Improved green fluorescence. Nature.

[9] Heim R., Prasher D.C., Tsien R.Y. (1994). Wavelength mutations and posttranslational autoxidation of green fluorescent protein. Proc. Natl. Acad. Sci. USA.

[10] Ma Y., Sun Q., Zhang H., Peng L., Yu J.-G., Smith S.C. (2010). The Mechanism of Cyclization in Chromophore Maturation of Green Fluorescent Protein: A Theoretical Study. J. Phys. Chem. B.

[11] Miyawaki A., Shcherbakova D.M., Verkhusha V. (2012). V Red fluorescent proteins: Chromophore formation and cellular applications. Curr. Opin. Struct. Biol..

[12] Pletneva N.V., Pletnev V.Z., Lukyanov K.A., Gurskaya N.G., Goryacheva E.A., Martynov V.I., Wlodawer A., Dauter Z., Pletnev S. (2010). Structural Evidence for a Dehydrated Intermediate in Green Fluorescent Protein Chromophore Biosynthesis. J. Biol. Chem..

[13] Verkhusha V.V., Chudakov D.M., Gurskaya N.G., Lukyanov S., Lukyanov K.A. (2004). Common Pathway for the Red Chromophore Formation in Fluorescent Proteins and Chromoproteins. Chem. Biol..

[14] Yum C., Ahn T., Shim W.-S. (2020). Development of a Novel Blue Fluorescent Gene-encoded Calcium Indicator Modified from GCaMP3. J. Fluoresc..

[15] Liebschner D., Afonine P.V., Baker M.L., Bunkóczi G., Chen V.B., Croll T.I., Hintze B., Hung L.-W., Jain S., McCoy A.J. (2019). Macromolecular structure determination using X-rays, neutrons and electrons: Recent developments in Phenix. Acta Crystallogr. Sect. D Struct. Biol..

[16] Akerboom J., Carreras Calderón N., Tian L., Wabnig S., Prigge M., Tolö J., Gordus A., Orger M.B., Severi K.E., Macklin J.J. (2013). Genetically encoded calcium indicators for multi-color neural activity imaging and combination with optogenetics. Front. Mol. Neurosci..

[17] Kremers G.-J., Goedhart J., van den Heuvel D.J., Gerritsen H.C., Gadella T.W.J. (2007). Improved Green and Blue Fluorescent Proteins for Expression in Bacteria and Mammalian Cells. Biochemistry.

[18] Mena M.A., Treynor T.P., Mayo S.L., Daugherty P.S. (2006). Blue fluorescent proteins with enhanced brightness and photostability from a structurally targeted library. Nat. Biotechnol..

[19] Subach O.M., Cranfill P.J., Davidson M.W., Verkhusha V.V. (2011). An Enhanced Monomeric Blue Fluorescent Protein with the High Chemical Stability of the Chromophore. PLoS ONE.

[20] Subach O.M., Malashkevich V.N., Zencheck W.D., Morozova K.S., Piatkevich K.D., Almo S.C., Verkhusha V.V. (2010). Structural Characterization of Acylimine-Containing Blue and Red Chromophores in mTagBFP and TagRFP Fluorescent Proteins. Chem. Biol..

[21] Acharya A., Bogdanov A.M., Grigorenko B.L., Bravaya K.B., Nemukhin A.V., Lukyanov K.A., Krylov A.I. (2017). Photoinduced Chemistry in Fluorescent Proteins: Curse or Blessing?. Chem. Rev..

[22] Bravaya K.B., Grigorenko B.L., Nemukhin A.V., Krylov A.I. (2012). Quantum Chemistry Behind Bioimaging: Insights from Ab Initio Studies of Fluorescent Proteins and Their Chromophores. Acc. Chem. Res..

[23] Khrenova M.G., Mulashkin F.D., Bulavko E.S., Zakharova T.M., Nemukhin A.V. (2020). Dipole Moment Variation Clears Up Electronic Excitations in the π-Stacked Complexes of Fluorescent Protein Chromophores. J. Chem. Inf. Model..

[24] Khrenova M.G., Mulashkin F.D., Nemukhin A.V. (2021). Modeling Spectral Tuning in Red Fluorescent Proteins Using the Dipole Moment Variation upon Excitation. J. Chem. Inf. Model..

[25] Winter G., Waterman D.G., Parkhurst J.M., Brewster A.S., Gildea R.J., Gerstel M., Fuentes-Montero L., Vollmar M., Michels-Clark T., Young I.D. (2018). DIALS: Implementation and evaluation of a new integration package. Acta Crystallogr. Sect. D Struct. Biol..

[26] Evans P. (2006). Scaling and assessment of data quality. Acta Crystallogr. Sect. D Biol. Crystallogr..

[27] Vagin A., Teplyakov A. (1997). MOLREP: An Automated Program for Molecular Replacement. J. Appl. Crystallogr..

[28] Murshudov G.N., Skubák P., Lebedev A.A., Pannu N.S., Steiner R.A., Nicholls R.A., Winn M.D., Long F., Vagin A.A. (2011). REFMAC 5 for the refinement of macromolecular crystal structures. Acta Crystallogr. Sect. D Biol. Crystallogr..

[29] Smart O.S., Womack T.O., Flensburg C., Keller P., Paciorek W., Sharff A., Vonrhein C., Bricogne G. (2012). Exploiting structure similarity in refinement: Automated NCS and target-structure restraints in BUSTER. Acta Crystallogr. Sect. D Biol. Crystallogr..

[30] Collaborative Computational Project N. (1994). 4 The CCP4 suite: Programs for protein crystallography. Acta Crystallogr. Sect. D Biol. Crystallogr..

[31] Emsley P., Cowtan K. (2004). Coot: Model-building tools for molecular graphics. Acta Crystallogr. Sect. D Biol. Crystallogr..

[32] Krissinel E., Henrick K. (2007). Inference of Macromolecular Assemblies from Crystalline State. J. Mol. Biol..

[33] Krissinel E., Henrick K. (2004). Secondary-structure matching (SSM), a new tool for fast protein structure alignment in three dimensions. Acta Crystallogr. Sect. D Biol. Crystallogr..

[34] Denning E.J., Priyakumar U.D., Nilsson L., Mackerell A.D. (2011). Impact of 2′-hydroxyl sampling on the conformational properties of RNA: Update of the CHARMM all-atom additive force field for RNA. J. Comput. Chem..

[35] Best R.B., Zhu X., Shim J., Lopes P.E.M., Mittal J., Feig M., MacKerell A.D. (2012). Optimization of the Additive CHARMM All-Atom Protein Force Field Targeting Improved Sampling of the Backbone ϕ, ψ and Side-Chain χ_1_ and χ_2_ Dihedral Angles. J. Chem. Theory Comput..

[36] Vanommeslaeghe K., Hatcher E., Acharya C., Kundu S., Zhong S., Shim J., Darian E., Guvench O., Lopes P., Vorobyov I. (2010). CHARMM general force field (CGenFF): A force field for drug-like molecules compatible with the CHARMM all-atom additive biological force fields. J. Comput. Chem..

[37] Jorgensen W.L., Chandrasekhar J., Madura J.D., Impey R.W., Klein M.L. (1983). Comparison of simple potential functions for simulating liquid water. J. Chem. Phys..

[38] Phillips J.C., Hardy D.J., Maia J.D.C., Stone J.E., Ribeiro J.V., Bernardi R.C., Buch R., Fiorin G., Hénin J., Jiang W. (2020). Scalable molecular dynamics on CPU and GPU architectures with NAMD. J. Chem. Phys..

[39] Adamo C., Barone V. (1999). Toward reliable density functional methods without adjustable parameters: The PBE0 model. J. Chem. Phys..

[40] Grimme S., Antony J., Ehrlich S., Krieg H. (2010). A consistent and accurate ab initio parametrization of density functional dispersion correction (DFT-D) for the 94 elements H-Pu. J. Chem. Phys..

[41] Melo M.C.R., Bernardi R.C., Rudack T., Scheurer M., Riplinger C., Phillips J.C., Maia J.D.C., Rocha G.B., Ribeiro J.V., Stone J.E. (2018). NAMD goes quantum: An integrative suite for hybrid simulations. Nat. Methods.

[42] TeraChem v 1.9, PetaChem, LLC. www.petachem.com.

[43] Lu T., Chen F. (2012). Multiwfn: A multifunctional wavefunction analyzer. J. Comput. Chem..

[44] Lu T., Chen F. (2013). Bond Order Analysis Based on the Laplacian of Electron Density in Fuzzy Overlap Space. J. Phys. Chem. A.

[45] Bader R.F.W., Slee T.S., Cremer D., Kraka E. (1983). Description of conjugation and hyperconjugation in terms of electron distributions. J. Am. Chem. Soc..

[46] Mardirossian N., Head-Gordon M. (2015). Mapping the genome of meta-generalized gradient approximation density functionals: The search for B97M-V. J. Chem. Phys..

[47] Neese F. (2012). The ORCA program system. Wiley Interdiscip. Rev. Comput. Mol. Sci..

